# Potential radiotherapy-related reactivation of immune checkpoint inhibitor hepatitis

**DOI:** 10.1007/s00066-024-02361-0

**Published:** 2025-02-04

**Authors:** Kakharman Yesmembetov, Cennet Sahin, Mohamad Murad, Marie-Luise Berres, Alexander Koch, Martin von Websky, Florian Vondran, Philipp Bruners, Michael Eble, Ahmed Allam Mohamed

**Affiliations:** 1 Gastroenterology, Hepatology and infectious Diseases Department, University Hospital RWTH Aachen, Aachen, Germany; 2 Visceral and Transplantation Surgery Department, University Hospital RWTH Aachen, Aachen, Germany; 3 Diagnostic and interventional Radiology Department, University Hospital RWTH Aachen, Aachen, Germany; 4 Radiation Oncology Department, University Hospital RWTH Aachen, Pauwelstraße 30, 52074 Aachen, Germany; 5 Center for Integrated Oncology Aachen, Bonn, Cologne and Duesseldorf (CIO ABCD), Aachen, Germany; 6 National research oncology center, Center for Hepatopancreatobiliary Surgery and Organ Transplantation, Kerey Zhanibek khandar street 3, Z05K4F3 Astana, Kazakhstan

**Keywords:** Immue checkpoint inhibitors, SBRT, Immune-related adverse events, Autoimmune hepatitis, Cholnagiocellular carcinoma

## Abstract

This report details the reactivation of immune checkpoint inhibitor (ICI)-related autoimmune hepatitis triggered by stereotactic body radiation therapy (SBRT) in a 55-year-old male with hilar cholangiocellular carcinoma. Initially diagnosed in December 2021, the patient underwent successful resection and subsequent adjuvant therapy. Despite stable disease following chemotherapy augmented with durvalumab, he developed grade 3 acute hepatitis after seven cycles of durvalumab. Following a brief prednisolone regimen and normalization of liver tests, SBRT targeting para-aortic lymph nodes was initiated. Remarkably, severe hepatitis reoccurred 7 days after starting SBRT, 88 days following the last durvalumab infusion, necessitating resumed and escalated prednisolone treatment. Another course of SBRT for a newly diagnosed metastatic liver lesion was administered in September 2023, with ongoing prednisolone adjustment. By February 2024, liver tests normalized, but subsequent radiological assessments indicated tumor progression, leading to the reintroduction of chemotherapy. This case underscores the potential of SBRT for activating severe immune-mediated hepatotoxicity in patients treated with ICIs, highlighting the need for careful monitoring and management of such patients. Further, this report highlights the possible survival benefit of the strategic application of SBRT in addition to systematic treatment in recurrent and metastatic cholangiocellular carcinoma.

## Background

Immune checkpoint inhibitors (ICIs) represent a transformative advance in oncology, offering new therapeutic avenues for various cancers. By targeting regulatory pathways of T lymphocytes, specifically the PD-1/PD-L1 and CTLA‑4 axes, ICIs boost the immune system’s ability to combat malignancies [1]. However, activating immune responses can sometimes result in unintended consequences, known as immune-related adverse events (irAEs), affecting multiple organ systems including the liver. Here, we present a compelling case of immune checkpoint inhibitor-related autoimmune hepatitis (ICH) triggered by stereotactic body radiation therapy (SBRT) in a patient with hilar cholangiocellular carcinoma.

The case underscores the intricate interplay between radiation and systemic immunotherapy, highlighting the potential for irAEs even with localized treatment modalities. As ICIs continue to be integrated into cancer management, understanding their synergistic and adverse interactions with other therapies becomes crucial. This case provides valuable insights into managing complex clinical scenarios involving advanced therapies and contributes to the broader understanding of ICI-related hepatotoxicity and its management.

## Case presentation

A 55-year-old male was initially diagnosed with hilar cholangiocellular carcinoma (Klatskin tumor) in December 2021. Upon confirmation of the absence of distant metastases, he underwent right-sided hepatectomy, resection of segment I, and bifurcation of the hepatic ducts, with the creation of a biliodigestive anastomosis. The postoperative stage was classified as G2 pT2a pN1 (3/12) L0 V0 Pn1 R0.

Following the R0 resection, adjuvant therapy with weight-adjusted capecitabine commenced in February 2022. However, after four cycles, the treatment shifted to a combination of gemcitabine and cisplatin due to lymphogenic recurrence involving the para-aortic lymph nodes in May 2022. From the fifth cycle in September 2022, chemotherapy was supplemented with durvalumab, aligned with findings from the TOPAZ‑1 clinical trial recently published at the time [2]. After confirming disease stability through eight cycles in December 2022, treatment continued with durvalumab alone at the patient’s request, to mitigate chemotherapy-related bone marrow and gastrointestinal toxicity.

**Fig. 1:**
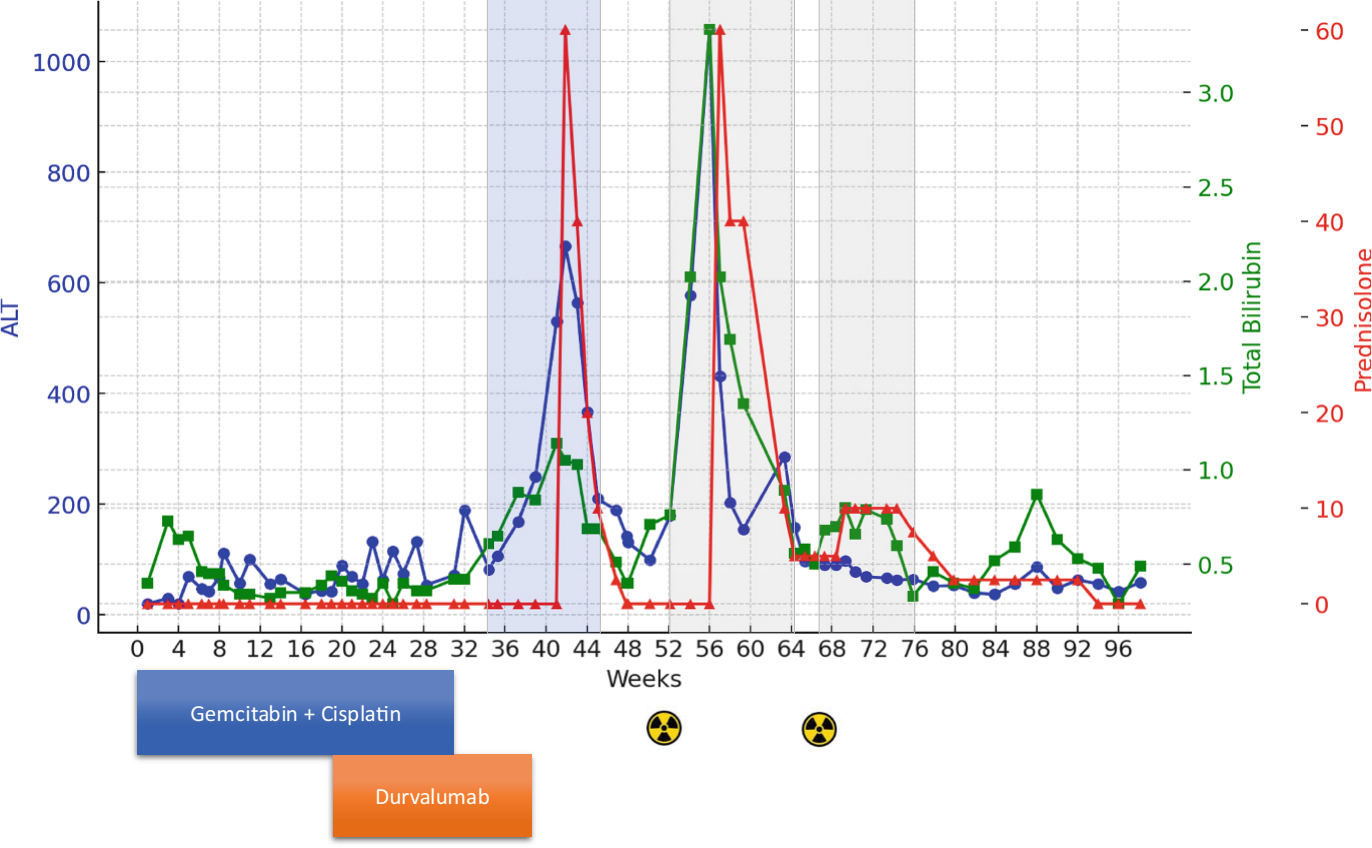
A timeline illustrates the sequence of events experienced by the patient during the recurrence of his disease, detailing the administration of chemotherapy (gemcitabine and cisplatin), immunotherapy (durvalumab), and radiation therapy (denoted with trefoil symbols). It includes graphs depicting the levels of Alanine transaminase (ALT) (blue), total bilirubin (green), and the dose of prednisolone in grams (red). Additionally, the timeline highlights the phases of immune-related adverse events (irAEs), primarily immune checkpoint inhibitor-related autoimmune hepatitis (ICH), as indicated in light blue. Reactivations of irAEs are marked in light grey

**Fig. 2:**
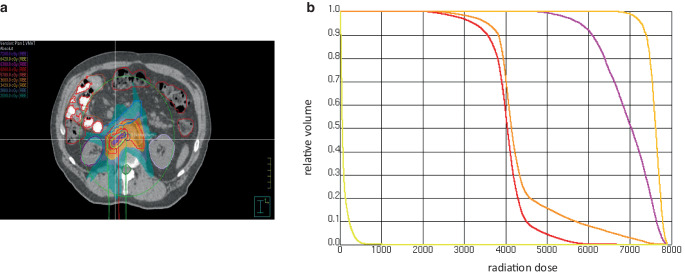
**a** Dose color wash from the patient’s stereotactic body radiotherapy (SBRT) plan targeting recurrent para-aortic lymph nodes. **b** Dose–volume histogram of the radiation plan: CTV and PTV of the metastatic node in light orange and purple, respectively; CTV and PTV of the elective nodal region in orange and red, respectively; and liver in yellow

In February 2023 (week 42 since recurrence), after seven cycles of durvalumab, the patient developed grade 3 acute hepatitis [3], with the following values: Alanine transaminase (ALT) 530 U/L (10.6x ULN), Aspartate transaminase (AST) 318 U/L (6.4x ULN), total bilirubin 1.14 mg/dL (normal), Alkaline phosphatase (AP) 385 U/L (3.0x ULN), and Gamma-glutamyltransferase (GGT) 382 U/L (6.4x ULN), as shown in Fig. 1. After ruling out other causes, including acute viral hepatitis, the case was diagnosed as an irAE, and a prednisolone regimen commenced 9 days later at 60 mg/day until improvement of ICH to grade 2 [4]. Prednisolone was gradually reduced over 5 weeks and discontinued by the end of March 2023, coinciding with the normalization of liver tests. Based on a multidisciplinary tumor board (MDT) recommendation, SBRT targeting the aforementioned para-aortic lymph nodes with 60 Gy in 12 fractions at 80% isodose to the metastatic nodes and elective nodal irradiation of the paraaortic lymphatic basin at 36 Gy in 12 fractions (Fig. 2a) began in April 2023 (week 51 since the recurrence) and was delivered over 3 weeks. The mean dose to the liver was 1 Gy, and the dose to 700 cm^3^ of liver volume was 0.97 Gy (Fig. 2b).

**Fig. 3:**
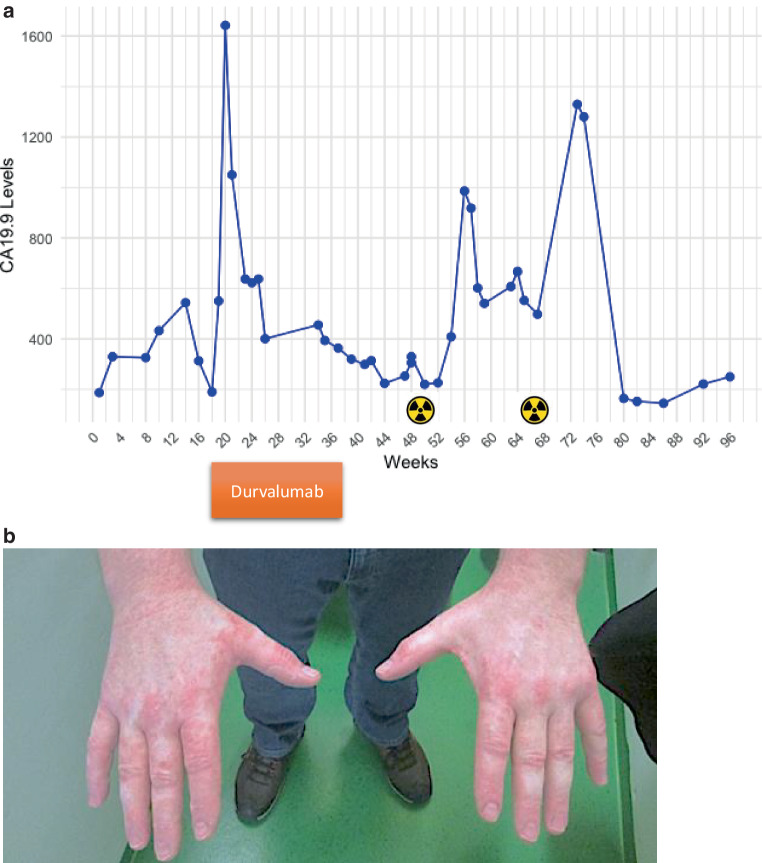
**a** Levels of CA19‑9 throughout the course of disease recurrence, showing increases following the initiation of durvalumab treatment and radiation therapies. **b** Photographic documentation of the skin rash developed following the patient’s SBRT treatment

Seven days after initiation of radiation (week 52), 88 days after the last durvalumab infusion, and 26 days after discontinuation of steroids, the patient exhibited escalating liver enzyme levels, culminating in grade 4 acute hepatitis 116 days after the last durvalumab infusion, with the following levels: ALT 1057 U/L (21.1x ULN), AST 648 U/L (13x ULN), total bilirubin 3.3 mg/dL (2.8x ULN), AP 375 U/L (2.9x ULN), and GGT 388 U/L (6.5x ULN), accompanied by a skin rash (Fig. 3a) and flare in CA 19.9 986 U/L (Fig. 3b). Following sonographic exclusion of cholestasis, prednisolone treatment resumed at 1 mg/kg/day due to a resurgence of ICH triggered by SBRT. In response to the rapid decline in liver test values, the prednisolone dosage was tapered to 5 mg/daily. Over the subsequent weeks, the prednisolone dosage was adjusted and maintained within the range of 5 to 10 mg/day, guided by the liver tests.

**Fig. 4:**
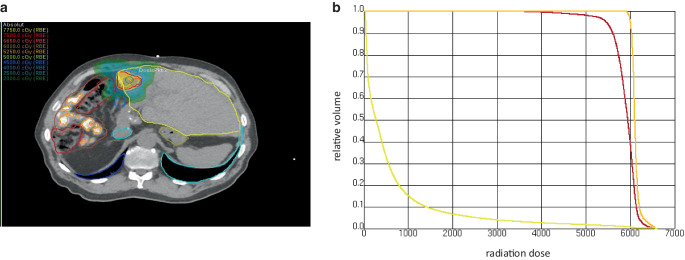
**a** Dose color wash from the patient’s stereotactic body radiotherapy (SBRT) plan for treating liver metastases. **b** Dose–volume histogram of the radiation plan: CTV and PTV of the liver metastatic node in light orange and red, respectively, and liver in yellow

In September 2023, the patient received another SBRT course, delivering 50 Gy in five fractions prescribed to isodose line 80% around the planning tumor volume for a newly diagnosed metastatic liver lesion (Fig. 4a); the mean dose to the liver was 6 Gy and the dose of 700 cm^3^ of the liver volume was 4.18 Gy (Fig. 4b). The prednisolone dosage was prophylactically maintained at 10 mg/day during the 2‑week radiation period to avoid a third episode of ICH. Nevertheless, the patient developed a mild skin rash on his arms and face at the end of the radiation and a flare in CA19.9 (week 73: 1330 U/L) (Fig. 3a). Subsequently, prednisolone was gradually tapered off and wholly discontinued in February 2024, aligning with normalized liver tests. Radiological evaluations in February 2024 showed no evidence of tumor lesions. Unfortunately, the patient exhibited tumor progression by April 2024 with new liver metastases, prompting the reintroduction of gemcitabine and cisplatin therapy.

## Discussion

While ICIs effectively disinhibit PD-1/PD-L1 and CTLA‑4 pathways to attack cancer cells, they can also potentially disrupt immune homeostasis, leading to irAEs. ICH is one notable irAE, observed in approximately 5–10% of patients on ICI monotherapy and at a significantly higher rate of 25–30% in those receiving combined anti-PD(L)1–anti-CTLA-4 therapy [5]. In the context of the TOPAZ-1 trial, irAEs occurred in 12.7% of patients, with ICH comprising 1.2% of these cases, illustrating that while these events are uncommon, they are not rare and require vigilance due to their potential severity [2].

The etiology of ICH is complex and multifaceted, involving several mechanisms. These include the direct cytotoxic effects of antibodies, autoimmune reactions due to epitope spreading, a breakdown in regulatory T‑cell-mediated tolerance, and liver toxicity driven by TNF‑α [5]. The liver’s distinct immunological characteristics, particularly its capacity to either activate or promote tolerance in CD8+ T cells and its role in anchoring these cells during immune responses [6], may also play a crucial role in developing ICH.

The management of ICH is based on its severity [3]. Grade 1 requires only monitoring of liver enzymes, while grade 2 necessitates withholding ICI therapy and initiation of corticosteroids in case of rising transaminases. Grade 3 ICH requires stopping ICI therapy, intravenous corticosteroids 1 mg/kg, and consideration of inpatient care and liver biopsy. Grade 4 ICH necessitates permanent stopping of ICI and i.v. methylprednisolone 2 mg/kg. For both grades 3 and 4, gradual tapering of corticosteroids over a period of 4 weeks is recommended once the condition improves to grade 2.

Radiation therapy (RT) may amplify the body’s immune response against tumors through various mechanisms. These include promoting the maturation and activation of dendritic cells, enhancing MHC class I expression and tumor antigen presentation, and fostering the infiltration of effector immune cells like CD8+ T cells into the tumor microenvironment [7]. The abscopal effect, a rare but notable phenomenon, occurs when localized radiation therapy induces regression in distant, non-irradiated metastatic tumors, believed to be mediated by the immune system being activated by the treatment. This effect is particularly potentiated when RT is combined with immunotherapy due to radiation exposing tumor antigens and immunotherapy inhibiting immune response suppressors.

Additionally, “radiation recall” is another phenomenon observed with RT, characterized by an inflammatory response in previously irradiated tissues reactivated by systemic agents like chemotherapy or immunotherapy [8]. Though typically associated with chemotherapy, recent findings suggest that ICIs can also trigger similar inflammatory reactions, such as dermatitis, pneumonitis, and mucositis [9–12]. The underlying mechanisms for radiation recall reactions are not fully understood but involve acute or chronic inflammation marked by cellular infiltration and vascular changes [8].

Our case extends the spectrum of previously mentioned phenomena by demonstrating a recall of ICI toxicities precipitated by radiotherapy, specifically manifesting as severe hepatitis with a flare in CA19.9 and dermatitis. This suggests a complex interplay between radiotherapy and residual immune alterations induced by ICIs, with possible activation of autoimmunity.

Weng et al. described a similar phenomenon; however, it included the reactivation of ICI nephritis after starting chemoradiation [13]. To the best of our knowledge, the recall of ICI hepatitis by radiation after discontinuing the ICI has not been previously reported. However, a common point between the two reports is the ability of RT to reactivate mainly the previously reported irAEs.

Although there was a 26-day interval between the discontinuation of steroids and the development of reactivated ICH and a longer timeline preceding the second reactivation of other irAEs, the possibility that these reactivations were remotely influenced by steroid discontinuation or dosage adjustments cannot be entirely excluded.

In recent years, several prospective clinical trials, including EORTC-1560-GITCG, have demonstrated the feasibility and safety of combining liver stereotactic body radiotherapy (SBRT) with immune checkpoint inhibitors such as PD-1/PD-L1 inhibitors [14–16]. These studies have shown promising results, with the combination proving effective in enhancing local tumor control while maintaining an acceptable toxicity profile. SBRT, when combined with PD-1/PD-L1 inhibitors, is thought to potentiate the immune-mediated response against tumors by increasing tumor antigen presentation and promoting the infiltration of immune cells [17]. While the combination is generally considered safe, as highlighted by trials like EORTC-1560-GITCG, our case represents a rare instance of severe immune-related adverse events, specifically ICI-induced hepatitis potentially reactivated by radiotherapy.

Understanding these interactions fully could improve the efficacy of combining RT and ICIs and inform strategies to mitigate severe irAEs, thereby enhancing patient safety as ICIs become increasingly prevalent in cancer treatment.

Despite challenges, the strategic incorporation of SBRT alongside systemic treatments, including ICIs, for managing advanced or recurrent cholangiocellular carcinoma appears promising. In our current case, the patient achieved a survival of at least 26 months from the diagnosis of recurrence, demonstrating a significant improvement compared to the reported standard of care, which has a median overall survival of 12.9 months and a 24-month survival rate of 10.9% [2].
